# Towards a computational model of learning and social interactions of mice in IntelliCage

**DOI:** 10.1186/1471-2202-14-S1-P238

**Published:** 2013-07-08

**Authors:** Jakub M Kowalski, Anna Kiryk, Leszek Kaczmarek, Daniel K Wójcik, Szymon Łęski

**Affiliations:** 1 Department of Neurophysiology, Nencki Institute of Experimental Biology, Warsaw, 02-093, Poland; 2 Neurobiology Center, Nencki Institute of Experimental Biology, Warsaw, 02-093, Poland; 3 Department of Molecular and Cellular Neurobiology, Nencki Institute of Experimental Biology, Warsaw, 02-093, Poland

## 

In [1] we have studied cognitive deficits in transgenic mouse model of Alzheimer's Disease. In particular, we used automated mice cages (IntelliCage) to investigate how healthy and transgenic mice learn the location of a reward (sweetened water provided in only one of the four corners of the cage) in different social situations. We showed that co-housing transgenic mice with healthy companions can alleviate learning deficits. We also showed that the ability to find the reward is modulated by the circadian rhythm.

A natural continuation of that study is to find specific mechanisms of learning and social interactions responsible for the observed patterns of behavior. To that end we now employ a collection of computational models of learning and decision making [2]. In the models we use the Rescorla-Wagner rule for the learned values of the actions supplemented by a model-specific decision-making rule.

**Figure 1:**
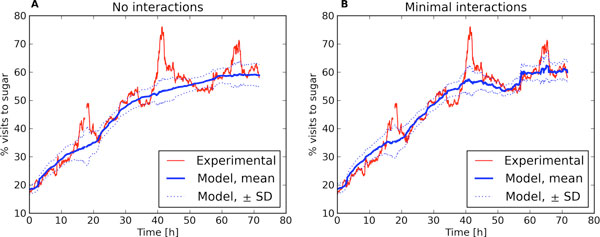
**Experimental and model learning curves of healthy mice**. Left: model without interactions between animals. Right: model with minimal social interactions. Thin lines mark the standard deviation of model curves over 100 repetitions.

In the simplest case we assume the decisions are based purely on the learned rewards, with the probabilities of actions given by the softmax distribution. Such a simple model, in which animals are ignorant of each other, fits the trend of the learning curve well both for healthy (Figure 1A) and transgenic (not shown) mice. However, it does not capture the circadian oscillations.

Next we study a model in which the interactions between animals are included in a minimal form. Specifically, the probability of visiting given corner is reduced if the corner is already occupied by another mouse at the time the choice of the action takes place. In this case we observe that the model learning-curve starts to exhibit oscillations around the trend (Figure 1B). Still, the amplitude of these oscillations is significantly smaller than in experimental data, which suggests that other effects come also into play.
